# Parasite Specific Antibody Levels, Interferon-γ and TLR2 and TLR4 Transcripts in Blood from Dogs with Different Clinical Stages of Leishmaniosis

**DOI:** 10.3390/vetsci5010031

**Published:** 2018-03-16

**Authors:** Sara Montserrat-Sangrà, Laura Ordeix, Pamela Martínez-Orellana, Laia Solano-Gallego

**Affiliations:** 1 Departament de Medicina i Cirurgia Animals, Universitat Autònoma de Barcelona, 08193 Bellaterra, Spain; sara.montserrat@uab.cat (S.M.-S.); laura.ordeix@uab.cat (L.O.); pamela.martinez@uab.cat (P.M.-O.); 2 Fundació Hospital Clínic Veterinari, Facultat de Veterinària, Universitat Autònoma de Barcelona, 08193 Bellaterra, Spain

**Keywords:** dog, *Leishmania infantum*, papular dermatitis, IFN-γ, TLR2, TLR4, blood parasitemia

## Abstract

Canine leishmaniosis has a wide range of disease severity from mild (stage I), to severe (stages II–III), or very severe disease (stage IV). The objective of the study was to evaluate and compare serum antibody levels, *Leishmania infantum* specific IFN-γ production and TLR2 and TLR4 transcripts in non-stimulated blood from dogs with different clinical stages at the time of diagnosis as well as blood parasitemia. Enzyme-Linked ImmunoSorbent Assay (ELISAs) were performed to determine serum antibody levels and IFN-γ production and quantitative polymerase chain reaction (*q*PCRs) in order to determine blood parasite load and TLR2 and TLR4 transcripts. Older dogs were significantly affected by more severe disease with higher antibody levels and blood parasitemia than dogs with mild disease. IFN-γ production was significantly higher in dogs with stage I disease when compared to dogs with more severe disease. Relative quantification of TLR2 in dogs with mild disease was similar to that of control dogs. On the other hand, TLR2 transcripts were significantly higher in dogs with severe disease as compared with that from control healthy dogs. No differences were found in TLR4 relative quantification between groups. This study demonstrates that dogs with different clinical stages of leishmaniosis present different levels of biological markers indicative of different immune responses.

## 1. Introduction

Canine leishmaniosis (CanL) due to *Leishmania infantum* is a very pleomorphic disease. Clinically, this infection ranges from subclinical infection to very severe disease, passing through several degrees of disease [1]. According to this clinical variability, a clinical staging system that classifies the disease into four stages based on clinical signs, clinicopathological abnormalities, and measurement of anti-leishmanial antibodies, was previously described [1].

Clinical and clinical-pathological findings observed in CanL are the consequence of complex interactions between *L. infantum* and the genetical and immunological background of the dog [1]. In fact, both innate and adaptive immune responses play a role in the outcome of *Leishmania* infection [2,3]. However, only the adaptive immune response has been extensively investigated in dogs. The balance between the protective T-helper (Th) 1 cellular response, which was associated with the cytokine interferon-gamma (IFN-γ), and the humoral immune response mediated by Th2 lymphocytes determines the clinical manifestation of the infection. A predominantly Th1 immune response is thought to provide resistance to the development of disease. On the other hand, a predominantly Th2 immune response correlates with antibody production and disease progression [2,4]. Although the innate immune response has been scarcely studied in leishmaniosis, recent data suggests that it is paramount for the ultimate outcome of *Leishmania* infection [2,5,6]. In this sense, toll like receptors (TLRs), which are one of the most important pattern recognition receptor family, are central in the early host defense against pathogen and activate adapter molecules after binding to their ligand. The activated cascade then leads to induction or suppression of genes that influence the inflammatory response [7]. 

Limited information is available regarding the role of TLRs in canine *L. infantum* infection. Although, the exact role of TLRs in the pathogenesis of CanL has not been fully addressed, it would seem that there is an association between TLR2 and its pathogenesis [2]. In fact, it has been recently revealed that TLR2 upregulation in blood and skin seems to be associated with disease progression in dogs [2,8], and a reduction in TLR2 transcription has been described with treatment and clinical improvement [8]. Moreover, there is a lower expression of TLR2 in skin biopsies from dogs with mild disease (papular dermatitis) when compared with dogs with moderate or severe disease [9]. TLRs other than TLR2 have been scarcely investigated in experimental or natural *L. infantum* infection in dogs [10]. Regarding TLR4, it has been described that transcription of this TLR appears to be similar among dogs with clinical leishmaniosis and healthy seronegative dogs at the time of diagnosis with no changes during treatment follow-up, indicating a less important role of this TLR in clinical leishmaniosis [8].

Cutaneous lesions are the most common clinical signs in CanL [11,12] and they are clinically classified as typical (i.e., exfoliative dermatitis, ulcerative dermatitis of the bony prominences, onychogryphosis, and, in an endemic area, papular dermatitis) or atypical (i.e., muco-cutaneous nodular dermatitis, other ulcerative muco-cutaneous dermatitis than that mentioned above, sterile pustular dermatitis, or ischaemic dermatopathy) [12]. Among the cutaneous manifestations of CanL, papular dermatitis is considered to be a typical form in an endemic area and is indicative of stage I leishmaniosis [13,14,15]. In fact, dogs with papular dermatitis commonly are young dogs without any other clinical-pathological abnormalities and with low parasite load and granuloma formation in skin lesions, negative or weakly positive anti-*Leishmania* antibody levels and specific cell-mediated immune response studied by means of leishmanin skin test [13,14,15]. Furthermore, this dermatological problem is associated with the spontaneous resolution of the lesions within 3–5 months [16]. Moreover, normal-looking skin from dogs with stage I and papular dermatitis is less likely to present microscopic lesions as well as harbour the parasite when compared with dogs with moderate to severe CanL [14]. Taken all of these findings together, papular dermatitis and stage I CanL is indicative of a protective immune response associated with a good prognosis [13,16,17]. However, there are limited studies that evaluate differences in clinical staging and adaptive and innate immune responses in CanL [18].

The hypothesis of this study is that dogs with stage I leishmaniosis and papular dermatitis show distinctive immunological characteristics when compared with dogs with more severe disease. Therefore, the objective of this study was to evaluate serum antibody levels, *L. infantum* specific IFN-γ production, and TLR2 and TLR4 transcripts in the blood of dogs with different clinical stages of leishmaniosis at the time of diagnosis.

## 2. Materials and Methods

### 2.1. Dogs

All of the dogs used in this study were privately owned pets, which were volunteered with client informed consent. They remained under the care of their owners and were not housed for the purposes of this study or held for any period of time. 

### 2.2. Dogs with Clinical Leishmaniosis

Forty-two dogs with typical cutaneous signs due to leishmaniosis attending different veterinarian facilities from Catalonia (Spain): Fundació Hospital Clínic Veterinari, Universitat Autònoma de Barcelona (UAB), Cerdanyola, Barcelona), Hospital Ars Veterinària (Barcelona), Mediterrani Veterinaris Hospital (Reus, Tarragona), and Consultori Montsant (Falset, Tarragona), were prospectively enrolled. In order to evaluate its clinical status, a full complete blood count (CBC), serum biochemical profile which included creatinine, urea, total cholesterol, total protein and alanine aminotransferase (ALT), protein serum electrophoresis, and urinalysis with urinary protein creatinine ratio (UPC) were carried out. The hematology, biochemistry, and serum electrophoresis were performed using the following laboratory equipment: Siemens ADVIA120 (Siemens Healthineers, Erlangen, Germany), chemistry analyzer Olympus AU400 (Olympus Diagnostica GmbH, Hamburg, Germany) and Hydrasis system (Sebia Hispania SA, Barcelona, Spain), respectively.

Leishmaniosis was diagnosed after visualization of amastigotes on skin cytology or biopsy with or without *Leishmania* specific immunohistochemistry, as previously described [9], and/or the detection of specific *L. infantum* antibody levels by quantitative serology using an in-house Enzyme-Linked ImmunoSorbent Assay (ELISA) [19]. The classification of *L. infantum* antibody levels is described in Supplementary Table S1. Dogs were classified based on LeishVet clinical stages, as previously described [1]. In addition, blood DNA extraction and *Leishmania* quantitative polymerase chain reaction (*q*PCR) was performed with an absolute quantification of parasites/mL of blood, a standard curve was created. Ten-fold serial dilutions of known concentrations were made from a culture with 10^7^ *L. infantum* promastigotes/mL. Six serial dilutions were obtained to create a standard curve. Subsequently, the value of the slope (m) and the intersection (y) of the line were calculated. The following formula was used in order to determine the number of parasites/mL blood by PCR: (Standard deviation − y)/m. The result was multiplied by the dilution factor of DNA (x125) to achieve the number of parasites/mL [20]. The classification of parasite load, as described elsewhere [21], is listed in Table S2.

### 2.3. Control Healthy Dogs

A total of 34 healthy dogs were enrolled. Both sexes were represented with 16 females and 18 males. Median of age was four years, with a range from six months to 10 years. Twenty-two purebred dogs with 14 breeds were represented (golden retriever, beagle, greyhound, Labrador retriever, border collie, schnauzer, Ibizan hound, fox hound, Ariege pointer, griffon Nivernais, Bruno Jura hound, Dachshund, English setter), eight mixed-breed dogs were included and for four dogs breeds were not registered. All of the dogs were seronegative and blood *q*PCR negative.

### 2.4. Whole Blood Cytokine Release Assay and Determination of Canine IFN-γ

Heparinized whole blood cytokine release assay was carried out, as previously reported [22]. Briefly, heparinized whole blood was separately mixed with three different conditions: (i) only medium (unstimulated); (ii) medium with soluble *L. infantum* antigen (LSA) at a concentration of 10 μg/mL; and, (iii) medium with the mitogen concanavalin A (ConA, 100 mg, Medicago^®^, Uppsala, Sweden) at a concentration of 10 μg/mL. IFN-γ was determined in supernatants from five days after stimulation with ConA and LSA or only medium (unstimulated), as described elsewhere [22], by a commercial sandwich ELISA (DuoSet^®^ ELISA by Development System R&DTM, Abingdon, UK).

Cytokine concentration from supernatants with ConA and LSA was calculated after subtracting the IFN-γ concentration obtained from supernatants with only medium (unstimulated). Thereafter, dogs were classified as IFN-γ producers when *L infantum* specific IFN-γ concentration was detectable. Dogs were classified as IFN-γ non-producers when *L. infantum* specific IFN-γ concentration was at not detectable levels [22]. 

### 2.5. RNA Extraction, RNA Concentration and Quality and cDNA Synthesis

Half mL of Ethylenediaminetetraacetic Acid (EDTA) blood sample was transferred to a cryovial tube that contained 1.3 mL of RNAlater (Ambion^®^, Thermo Fisher Scientific, Waltham, MA, USA). Then, blood samples in cryovial tubes were left at 4 °C overnight and, thereafter, frozen at −80 °C until used. Blood samples were thawed on ice and total RNA from 500 μL of EDTA blood was extracted using Ribopure RNA blood kit (Ambion^®^, Thermo Fisher Scientific), according to the manufacturer's instructions. A DNase digestion step was included to remove contaminating genomic DNA using TurboDNase (Ambion^®^, Thermo Fisher Scientific) following manufacturer’s instructions. RNA concentration was determined by Nanodrop device (Thermo Fisher Scientific). RNA integrity and quality was assessed by using an Agilent 2100 Bioanalyzer (Agilent Technologies, Santa Clara, CA, USA). Samples had a final concentration amongst 20–70 ng RNA/μL. All samples had an RNA Integrity Number (RIN) value greater than 7. cDNA was generated using VILO masterscript retrotranscriptase (Invitrogen™, Thermo Fisher Scientific), according to the manufacturer’s instructions. cDNA was stored at −20 °C until used [8].

### 2.6. TLR2, TLR4 and Reference Housekeeping Genes qPCR

Transcription of TLR2 and TLR4 target genes (TG), as well as three reference housekeeping genes (RG: HPRT-1, CG14980 and SDHA), was measured by *q*PCR using QuantStudio™ 7 Flex Real-Time PCR System, array card, desktop (Life Technologies, Carlsbad, CA, USA) as previously described [8]. Briefly, we calculated the efficiency of target and reference amplification genes and determined optimal efficiencies amplifications of 100% and acceptable in the range (±20%). The baseline and threshold were established for each gene and all of the samples were processed in triplicate. The same control sample was used as a calibrator in every plate to determine relative quantification of all the dogs studied. Data were processed while applying the relative quantification method comparable to the delta-delta-cycle threshold value (ddCt)-method (2^dd^Ct) [8]. Quantitative PCR data analysed was done by the Cloudsuite software (Life technologies™, Thermo Fisher Scientific). For normalization of TG, expression the arithmetic mean of the three RG was taken for the calculation of a reference gene index (RGI) [8]. 

### 2.7. Statistical Analysis

Statistical analyses were performed using the IBM SPSS Statistics Base 22.0 program for Windows software (IBM Corporation, Armonk, NY, USA).

Standard descriptive statistics [median and range (minimum and maximum values)] were calculated for quantitative variables. The non-parametric Mann-Whitney test was used to evaluate differences among groups. Categorical data were expressed as percentage and statistical analysis was performed while using the Fisher’s exact test to compare results among independent variables. Differences were considered significant at a 5% significance level (*p* < 0.05).

## 3. Results

### 3.1. Dogs with Clinical Leishmaniosis

**Figure 1:**
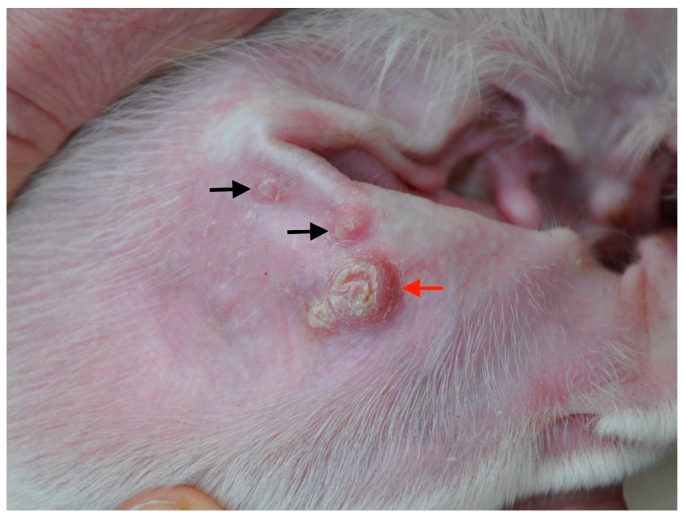
Two raised solid lesions smaller than 0.5 cm in diameter (papules, black arrows) and one raised solid lesion wider than higher (plaque, red arrow) in the inner aspect of a pinna of an Ibizan Hound dog with papular dermatitis and stage I leishmaniosis (Group A).

**Figure 2:**
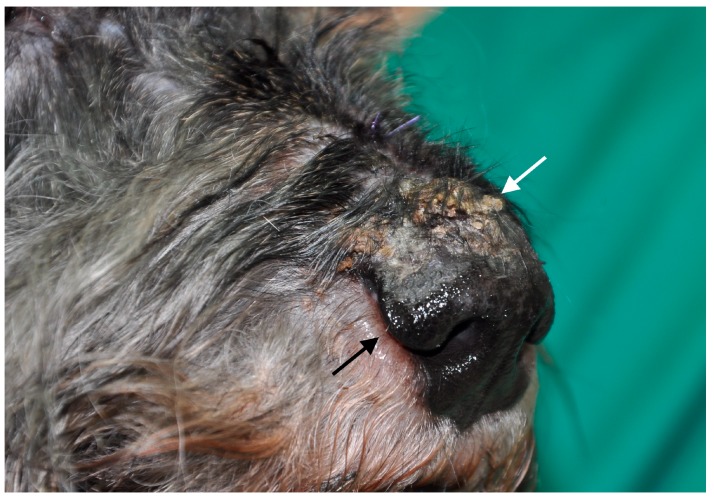
Crusting and scaling in the dorsal part of the muzzle (white arrow) and perinasal depigmentation (black arrow) in a schnauzer dog with stage II leishmaniosis (Group B).

Based on clinicopathological findings and *L. infantum*-specific antibody levels, dogs were clinically classified, as previously reported [1], and were distributed as follows: group A (stage I and papular dermatitis, *n* = 20, Figure 1) and group B (stage II–III and ulcerative or exfoliative dermatitis, *n* = 22, Figure 2). 

Ten females and ten males composed group A. Median of age was 10 months (5–98). Twelve pure bred dogs with three breeds represented and eight mixed breed dogs were included. Ten out 20 dogs were Ibizan hound dogs, one was a German pointer, and one was a Dachshund. Ten females and 12 males constituted group B. Median of age was 52.5 months (8–153). Fifteen pure bred dogs with 12 breeds represented (two French bull dogs, two Labrador retrievers, and one of each doberman, pinscher, American bull dog, schnauzer, boxer, Akita inu, greyhound, Brittany) and seven mixed breed dogs were included. The difference of age between groups was statistically significant (Mann-Whitney U-test, Z = 3.66999, *p* = 0.00024). Dogs from group A did not present laboratory abnormalities, while dogs from group B presented typical laboratory abnormalities of clinical leishmaniosis.

### 3.2. Blood Leishmania qPCR

Based on *Leishmania q*PCR dogs from group A were classified as negative (*n* = 12), low positive (*n* = 3), or medium positive (*n* = 1). The parasite load evaluation was not possible in four dogs from this group due to lack of sample. On the other hand, dogs from group B were classified as negative (*n* = 7), low (*n* = 9), medium (*n* = 4) and high positive (*n* = 2). A higher percentage of positive *Leishmania q*PCR in blood were found in group B when compared with group A (Fisher´s exact test, *p* = 0.0201).

Group A dogs had lower parasite load (median and range (R): 0 parasite/mL (0–33.49)) than those from group B (median and R: 2.54 (0–475.07); Mann-Whitney U-test, Z = −2.664, *p* = 0.012).

### 3.3. Leishmania infantum Specific Antibody Levels 

Dogs from group A were serologically classified as follows: negative (*n* = 11), very low positive (*n* = 3), low positive (*n* = 4), and medium positive (*n* = 2). On the other hand, dogs from group B were classified as very low positive (*n* = 1), low positive (*n* = 4), medium positive (*n* = 12), high positive (*n* = 3), and very high positive (*n* = 2). A higher percentage of seroreactive dogs was found in group B when compared with group A (Fisher´s exact test, *p* = 0.0001).

Group A dogs had lower levels of antibodies (median and R: 25 (5.7–529.5) EU) than those from group B (median and R: 1609 (86.8–90730.2) EU); Mann-Whitney U-test, Z = 3.53323, *p* = 0.00042).

### 3.4. Leishmania infantum Specific IFN-γ Production after Blood Stimulation

Cytokine analysis was performed in 19 out of 20 dogs from group A and in 15 out of 22 dogs from group B due to lack of samples. Fifteen of 19 dogs (79%) from group A and six out 15 (40%) from group B were classified as IFN-γ producers (Fisher exact test, *p* = 0.0337).

The median and R of IFN-γ production from groups A and B were 710 (0–20300) pg/mL and 0 (0–2413) pg/mL, respectively. There was a higher IFN-γ production in group A dogs when compared to those of group B (Mann-Whitney U-test, Z = 2.45047, *p* = 0.0143).

### 3.5. TLRs Transcripts 

**Figure 3:**
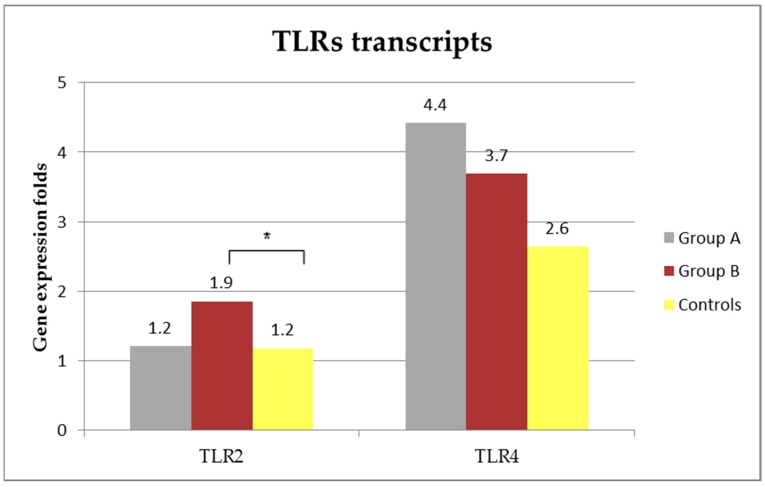
Median of Toll like receptors (TLR)2 and TLR4 transcripts in non-stimulated blood from diseased and control dogs (* Mann-Whitney U-test, Z = −2.41119, *p* = 0.016).

TLRS transcripts are presented in Figure 3. Relative quantification of TLR2 in group A (median and R: 1.2 (0.6–4.2) folds) was similar than that of control dogs (median and R: 1.2 (0.1–3.2) folds, Mann-Whitney U-test, Z = −0.24897, *p* = 0.8). On the other hand, relative quantification of TLR2 in group B (median and R: 1.9 (0.9–12.4)) was significantly higher when compared with that from control healthy dogs (Mann-Whitney U-test, Z = −2.41119, *p* = 0.016). Although no statistically significant, the expression of TLR2 in the group A was lower than that of the group B (Mann-Whitney U-test, Z = 1.6225, *p* = 0.1). 

The expression of TLR4 mRNA in control dogs was of a median and R of 2.6 (0.5–13) folds and was similar to that of dogs from group A (median and R: 4.4 (0.7–19.8) folds; Mann-Whitney U-test, Z = −0.92475, *p* = 0.36) and to that from group B (median: 3.7 (1.1–32.8) folds); Mann-Whitney U-test, Z = −1.88358, *p* = 0.06). Although not statistically significant, TLR4 mRNA expression in dogs with leishmaniosis was higher than that of control group. 

## 4. Discussion

The results of the present study support our hypothesis that dogs with stage I leishmaniosis and papular dermatitis show distinctive immunological characteristics when compared with dogs with more severe disease at the time of diagnosis. 

Dogs with stage I and papular dermatitis reported herein were more frequently serologically negative than the dogs with stage II–III. Moreover, in agreement with a previous study [14], we demonstrated that dogs with stage I and papular dermatitis had significantly lower levels of *Leishmania* antibodies than dogs with more severe disease. Clinical CanL is often associated with a humoral response (Th2 driven), which is non-protective and denotes failure to control the infection. Serologically negative and/or low positive dogs studied herein were further diagnosed by means of skin cytology, histopathology, and/or PCR. It is well established that dogs with mild clinical signs, such as solitary lymphadenomegaly or papular dermatitis, may present with negative to low positive antibody levels [23], as described in the present study. Therefore, lack or low humoral immune response elicited in dogs with stage I and papular dermatitis, may be indicative of a polarized specific cellular immune response (Th1 driven), which might confer protection against disease progression. 

In the present study, dogs with stage I and papular dermatitis were more commonly IFN-γ producers than dogs with more severe disease. In addition, *Leishmania*-specific IFN-γ concentrations were superior in dogs with papular dermatitis than in dogs with clinically more severe cutaneous forms. However, it is noteworthy that four dogs with mild disease were IFN-γ non–producers and that six dogs with more severe disease were IFN-γ producers. The results of the present study are in agreement with previous studies, which have shown that IFN-γ non-producers are usually classified in a more severe clinical staging than IFN-γ producers [18], and that IFN-γ concentration increase with long-term anti-*Leishmania* treatment, together with clinical improvement in dogs that do not produce IFN-γ at diagnosis [22]. Although, limited information regarding *L. infantum* specific IFN-γ production in stimulated blood in sick dogs is available, it seems that this assay is a reliable method of measurement of T cell-mediated immunity in CanL [24] and human leishmaniosis [25]. It is well established that Th1 lymphocytes mainly drive IFN-γ production in stimulated blood. However, natural killer (NK) cells, which play important roles in innate immune responses, may secrete this cytokine in response to IL-12. NK cells constitute 5% to 15% of the mononuclear cells in the blood. Therefore, the proportion of IFN-γ detected in blood from this origin is likely to be low. Identification of circulating cellular subsets expressing this immune marker was not carried out in the present study; therefore IFN-γ production cannot be conclusively attributed to an adaptive immune response [26]. A presumed T cellular immune response in dogs with stage I and papular dermatitis, is, therefore, thought to provide resistance to the progression of disease in those patients. In fact, previous published data have demonstrated a good clinical outcome with spontaneous resolution and an apparent lack of disease progression in dogs affected of papular dermatitis and stage I [16,17]. Moreover, a recent study have revealed a lack of disease progression in a cohort of untreated dogs with papular dermatitis and stage I leishmaniosis, followed during one year after the diagnosis [15]. However, it is interesting to highlight that those four IFN-γ non-producer dogs with papular dermatitis and stage I leishmaniosis would deserve a close and strict follow-up. 

Another limitation of this study is that cytokine immune profile related to Th2 immune response (i.e., TGF-β, IL10 or IL-4) was not evaluated in these dogs. Analysis of these cytokines in stimulated blood from dogs with leishmaniosis has been scarcely described, although an association was found between parasite specific IL-10 production and disease progression [27]. Using the same method that as employed in the present study, *L. infantum* specific IL-10 production after blood stimulation did not seem to be a disease severity marker since it was not detected in 64% of the patients and in those that was detected there was no correlation with the severity of the disease [22]. Therefore, although polarized Th1 immune response is strongly suggested in dogs with stage I and papular dermatitis, a Th2 like cytokine immune profile has not been ruled out in the present study.

There are limited data available on expression of TLRs in CanL. Therefore, the role of TLRs in the pathogenesis of this disease has not been fully addressed [10,28,29,30,31]. This study demonstrates that dogs with stage I and papular dermatitis presented a TLR2 transcription in the non-stimulated blood that was similar to healthy control dogs. On the other hand, there was a significantly higher up regulation of TLR2 in non-stimulated blood from dogs with severe clinical leishmaniosis at the time of diagnosis when compared with healthy control dogs. Although not being statistically significant, TLR2 transcripts were lower in stage I and papular dermatitis than in dogs with clinically more severe CanL. Although the cell source of increase in the expression of TLR2 was not determined in the present study, it is likely that the main source of the upregulation of TLR2 was firstly neutrophils and secondly monocytes and NK cells. Neutrophils are the predominant white cells in whole blood in canines. In addition, it has been also demonstrated that TLR2 protein is easily detectable by flow cytometry on the canine peripheral blood granulocyte and monocyte cell surface, and is rarely present on lymphocytes [32]. This is similar to what is found in humans where lymphocytes do not express TLR2 in unstimulated blood [33]. Dogs with moderate clinical leishmaniosis have a high parasite load in several tissues, including the bone marrow [34,35], and this might be the reason for a major up regulation of TLR2 in unstimulated blood, as *Leishmania* lipophosphoglycans are recognized by TLR2 [36] and it is likely that other parasite ligands are also involved in TLR2 recognition of *Leishmania* [37]. In addition, myeloid hyperplasia is a common finding in bone marrow of sick dogs with leishmaniosis [38]. Neutrophils and monocytes are released from the bone marrow to the peripheral blood, and thereafter they migrate to tissues where they die. Neutrophils or monocytes do not recirculate in peripheral blood. So, we hypothesize that it is likely that an upregulation of TLR2 happens in the bone marrow of dogs with more severe disease due to high parasite loads [34,35]. This high expression of TLR2 remains in neutrophils when they circulate in the peripheral blood, while it does not occur in dogs with mild disease.

Our results in unstimulated blood are in agreement with a previous study that described higher expression of TLR2 by immunohistochemistry in skin biopsies from cutaneous lesions in dogs with at least moderate disease (LeishVet stage II) when compared with dogs with mild disease due to papular dermatitis [9]. When considering these facts, it would seem that the transcription of TLR2 is not compartmentalized between the dermal tissue and the blood in diseased dogs. TLR2 appears to be a marker of inflammation in dogs, as demonstrated in the present study and in other canine inflammatory diseases, such as inflammatory bowel disease, immunomediated or bacterial arthritis, canine sino-nasal aspergillosis, and lymphoplasmacytic rhinitis or pyometra [39,40,41,42]. Based on published information, TLR2 up regulation appears to be associated with moderate to severe disease, suggesting an active innate immune and proinflammatory responses due to the presence of a high *Leishmania* parasite load or cellular damage (endogenous damage-associated molecular patterns (DAMPs), also called alarmins) [43], as mentioned above. In a study from Brazil, a high parasite load was found along with increased frequency and expression of TLR2 in cells from the colon of sick dogs [29]. In addition, the up regulation of TLR-2 genes has been positively associated with parasite load in the skin of naturally infected dogs [44]. According to this, parasite load in papular dermatitis has been demonstrated to be lower than in more severe cutaneous CanL states [14]. Moreover, dogs with papular dermatitis and stage I leishmaniosis show less microscopic skin lesions and parasite load in clinically healthy skin than dogs with more severe disease [14]. Therefore, a lower up regulation of TLR2 in this profile of dogs may be associated with this lower *Leishmania* parasite load. In addition, dogs with papular dermatitis and stage I leishmaniosis described in this study presented a significantly lower blood parasitemia when compared with dogs with at least moderate disease. 

The present results showed that TLR4 transcript in non-stimulated blood is unchanged among the groups studied. This finding is in agreement with previous studies performed on CanL [8], in which TLR4 transcription was unchanged when compared with healthy dogs, and might indicate a mechanism of parasite immune system evasion or a less important role of this TLR in clinical CanL. This is in agreement with studies that were performed in cutaneous lesions due to *Leishmania braziliensis* in humans, which demonstrated that TLR2 is the most common TLR expressed during active disease mainly by macrophages while TLR4 is scarcely expressed [45].

Although TLRs represent an important component of the innate immunity, there are many other inflammatory factors involved in this initial immune response, such as immunity cells, cell-associated molecules other than TLRs, and soluble molecules. Therefore, further studies regarding the analysis of these other factors is necessary in order to elucidate how innate immune responses prime the adaptive immune responses in *L. infantum* infection in dogs. 

## 5. Conclusions

In conclusion, this study demonstrates that dogs with stage I and papular dermatitis present lower specific antibody levels and blood parasitemia and higher specific IFN-γ concentrations after blood stimulation than dogs with more severe disease. Moreover, TLR2 transcript in dogs with mild disease was similar to that of healthy control dogs. On the other hand, TLR2 transcript in the blood of dogs with at least stage II was significantly higher than in healthy control dogs. The results from this study show distinctive immune responses in dogs with CanL. Moreover, immunological characteristics that were observed in dogs with stage I and papular dermatitis, together with low bood parasitemia, are indicative of the control of *Leishmania* infection. 

## Supplementary Materials

The following are available online at http://www.mdpi.com/2306-7381/5/1/31/s1, Table S1: Two-fold serial dilution ELISA classification in sera sample, Table S2: Parasite load classification in whole blood.
