# Supplementary material for: Parasite Specific Antibody Levels, Interferon-γ and TLR2 and TLR4 Transcripts in Blood from Dogs with Different Clinical Stages of Leishmaniosis
Source: Vet Sci. 2018 Mar 16;5(1):31. doi: 10.3390/vetsci5010031 (PMC5876572; doi:10.3390/vetsci5010031)
Supplement: Supplementary file 1 [file vetsci-05-00031-s001.pdf]

veterinary sciences

Table S1. Two-fold serial dilution ELISA classification in sera sample.

	Negative	Very Low Positive	Low Positive	Medium Positive	High Positive	Very High Positive
L. infantum antibody levels (EU)	<35	35–100	101–500	501–9000	9001–40000	≥40000

ELISA units (EU).

Table S2. Parasite load classification in whole blood.

	Negative	Low Positive	Medium Positive	High Positive	Very High Parasite Load
L. infantum parasite load (parasites/mL)	0	<10	10–100	101–1000	>1000
